# Identification of *PgRg1-3* Gene for Ginsenoside Rg1 Biosynthesis as Revealed by Combining Genome-Wide Association Study and Gene Co-Expression Network Analysis of Jilin Ginseng Core Collection

**DOI:** 10.3390/plants13131784

**Published:** 2024-06-27

**Authors:** Sizhang Liu, Xiaxia Chen, Tianqi Zhao, Jinghui Yu, Ping Chen, Yanfang Wang, Kangyu Wang, Mingzhu Zhao, Yue Jiang, Yi Wang, Meiping Zhang

**Affiliations:** 1 College of Life Science, Jilin Agricultural University, Changchun 130118, China; lsz512411412@163.com (S.L.); chenxiaxiaeee@163.com (X.C.); zhaotianqi0419@outlook.com (T.Z.); jinghui-yu@foxmail.com (J.Y.); chenping201407@126.com (P.C.); kangyu.wang@jlau.edu.cn (K.W.); mingzhuzhao@jlau.edu.cn (M.Z.); jiangyue285431@163.com (Y.J.); 2 Research Center for Ginseng Genetic Resources Development and Utilization, Jilin Province, Jilin Agricultural University, Changchun 130118, China; 3 College of Chinese Medicinal Materials, Jilin Agricultural University, Changchun 130118, China; yfwang2014@163.com

**Keywords:** genome-wide association study, weighted gene co-expression network analysis, ginsenoside Rg1, Rg1 biosynthesis genes, genetic effect, ginseng

## Abstract

Ginseng, an important medicinal plant, is characterized by its main active component, ginsenosides. Among more than 40 ginsenosides, Rg1 is one of the ginsenosides used for measuring the quality of ginseng. Therefore, the identification and characterization of genes for Rg1 biosynthesis are important to elucidate the molecular basis of Rg1 biosynthesis. In this study, we utilized 39,327 SNPs and the corresponding Rg1 content from 344 core ginseng cultivars from Jilin Province. We conducted a genome-wide association study (GWAS) combining weighted gene co-expression network analysis (WGCNA), SNP-Rg1 content association analysis, and gene co-expression network analysis; three candidate Rg1 genes (*PgRg1-1*, *PgRg1-2*, and *PgRg1-3*) and one crucial candidate gene (*PgRg1-3*) were identified. Functional validation of *PgRg1-3* was performed using methyl jasmonate (MeJA) regulation and RNAi, confirming that this gene regulates Rg1 biosynthesis. The spatial–temporal expression patterns of the *PgRg1-3* gene and known key enzyme genes involved in ginsenoside biosynthesis differ. Furthermore, variations in their networks have a significant impact on Rg1 biosynthesis. This study established an accurate and efficient method for identifying candidate genes, cloned a novel gene controlling Rg1 biosynthesis, and identified 73 SNPs significantly associated with Rg1 content. This provides genetic resources and effective tools for further exploring the molecular mechanisms of Rg1 biosynthesis and molecular breeding.

## 1. Introduction

Ginseng (*Panax ginseng* Mey.), a perennial herbaceous medicinal plant in the Araliaceae family, is renowned as the king of herbs [1,2,3,4]. Due to its high medicinal value, it is widely utilized in clinical treatments and the production of health foods worldwide [5,6,7]. Ginsenosides, a class of important secondary metabolites in the *Panax* genus, are the primary active ingredients of ginseng, belonging to the triterpene compound family. The diverse pharmacological activities of ginsenosides are determined by the different types of triterpene molecules, glycosylation sites, and the quantity of various glycosyl groups [8]. Among them, ginsenoside Rg1 belongs to the triterpene saponins, with the highest content among the triterpene saponins, and it exhibits a wide range of pharmacological effects, mainly focusing on anti-inflammatory, antioxidant, immunomodulatory, neuroprotective, and cognitive enhancement functions [9]. As a triterpene saponin, Rg1 originates from protopanaxatriol (PPT) in ginseng. Its synthesis process involves two pathways: firstly, UGTPg71A53 catalyzes the glycosylation at the C-20 position of PPT, forming ginsenoside F1, and then UGTPg71A54 catalyzes the glycosylation at the C-6 position of F1, synthesizing ginsenoside Rg1; secondly, UGTPg71A54 catalyzes the glycosylation at the C-6 position of PPT, forming ginsenoside Rh1, and then UGTPg71A29 catalyzes the glycosylation at the C-20 position of Rh1, resulting in ginsenoside Rg1 [10,11,12,13,14,15]. In the process of Rg1 biosynthesis, although several UGT genes have been cloned, they are insufficient to reveal the complexity of Rg1 biosynthesis [16,17,18]. It is still necessary to identify, clone, and study important genes involved in this biosynthesis pathway, such as cytochrome P450 genes, other UGT genes, or transcription factors, to provide genetic resources and a theoretical basis for elucidating the biosynthesis of ginsenoside Rg1.

Currently, the identification of genes related to ginsenoside biosynthesis mainly relies on known key enzyme genes in the triterpene metabolic pathways of heterologous species, and their homologous sequences are used as candidate genes for screening in ginseng. However, this method cannot directly identify new genes related to ginsenoside biosynthesis from ginseng. Currently, quantitative trait locus (QTL) mapping and genome-wide association study (GWAS) are the primary methods for identifying new genes, which have been widely applied in crops such as rice [19], cotton [20], and maize [21]. However, ginseng lacks mapping populations and genetic maps, making it impossible to identify new genes through QTL methods. However, GWAS does not require mapping populations and genetic maps; it can directly utilize natural populations or germplasm resources for multi-trait gene mapping. With the significant reduction in the cost of genome and transcriptome sequencing and the continuous improvement of software for SNP identification at the whole-genome level and SNP-trait linkage calculation, GWAS method has been conducted on crops to identify candidate genes controlling target traits. In ginseng, the core germplasm resources of Jilin ginseng have been established, and this core germplasm resource has been studied using whole-genome SNP molecular markers and 16 types of ginsenosides [22]. This provides essential germplasm resource populations, SNP molecular markers, and ginsenoside content traits necessary for identifying ginsenoside-related genes using GWAS in ginseng. Although GWAS can directly utilize natural populations or germplasm resources for trait gene mapping, this method identifies a large number of genes within the mapping interval, necessitating the combination with other methods to accurately identify candidate genes controlling the target traits.

The identification of candidate genes controlling target traits is a prerequisite for gene cloning. In order to accurately and efficiently identify candidate genes controlling ginsenoside biosynthesis, this study integrated GWAS analysis with weighted gene co-expression network analysis (WGCNA), SNP-content association analysis, and gene co-expression network analysis. This enabled the identification of candidate genes involved in ginsenoside Rg1 biosynthesis and SNP molecular markers significantly associated with Rg1 content at the whole-genome level. The functionality of candidate genes in Rg1 biosynthesis was verified through MeJA regulation and RNAi. Systematic analysis of cloned Rg1 biosynthesis genes elucidated their impact and role in Rg1 biosynthesis, providing genetic resources and effective SNP molecular markers for exploring the molecular mechanisms of Rg1 biosynthesis, ginseng quality evaluation, ginseng genome selection, and gene breeding.

## 2. Materials and Methods

### 2.1. Databases

This study utilized five databases. Database I originated from the transcriptomes of the roots of 4-year-old plants of 344 cultivars from the Jilin ginseng core germplasm resource (NCBI/GEO 369 SRR23758499—SRR23758802 and SRR13131364—SRR13131405) [22]. This database includes the sequences of gene transcripts, expression levels of transcripts and genes, single nucleotide polymorphisms (SNPs), nucleotide insertions/deletions (InDels), and the content of ginsenoside Rg1. Database II consists of the genome sequences of Chinese ginseng [23]. Database III includes the transcriptomes of the roots from the 5-, 12-, 18-, and 25-year-old plants of same ginseng cultivar, encompassing the gene transcript sequences and their expression levels (NCBI/SRA: SRX1445580—SRX1445583) [23]. Database IV consists of the transcriptomes of 14 tissues from 4-year-old ginseng plants in the red fruit stage in Jilin Province, China, including leaf blade, fruit pedicel, arm root, fruit peduncle, main root cortex, stem, rhizome, leg root, leaflet pedicel, fruit flesh, leaf peduncle, main root epiderm, fiber root, and seed, with sequences of gene transcripts and their expression levels (NCBI/SRA: SRX1445566—SRX1445579) [23]. Database V includes the transcriptomes of ginseng adventitious roots treated with MeJA for various durations: 0 h (control) and 6 h to 120 h. This database includes the expression levels of gene transcripts and the corresponding content of mono-ginsenosides at each treatment time, including the Rg1 ginsenoside content [24].

### 2.2. Identification of Candidate Genes for Ginsenoside Rg1 Biosynthesis

#### 2.2.1. Genome-Wide Association Study (GWAS)

##### Statistical Analysis of Ginsenoside Rg1 Content in Core Germplasm Resource Population

The phenotype data of Rg1 content were organized using Excel 2016, and the Best Linear Unbiased Prediction (BLUP) of Rg1 content was calculated using the lme4 package in the R language. The BLUP values were then used as data for subsequent identification of candidate genes involved in Rg1 biosynthesis and analysis of genetic effects. Frequency histograms of the calculated results were plotted using Origin 2022, and normality tests (Kolmogorov–Smirnov test, K-S test) were performed using IBM SPSS Statistics. The formula for blup calculation is as follows:

*blup* = *lmer* (*Rtaits* ~ (1|*ips*) + (1|*rep*)).

Here, blup is the Best Linear Unbiased Prediction; lmer, as a function for linear mixed-effects models, is used to fit linear mixed-effects models; Rtaits is the response variable, i.e., the trait data to be analyzed. Here, it is the response variable of the model. The random effect term indicates that ips is a random effect factor. Ips represents different individuals (ips: individuals/plants/strains); in plant systems, this usually refers to specific individuals, varieties, or genotypes. It represents individual differences under different genetic backgrounds. rep (replications/blocks): In experimental design, rep can represent different replicate trials.

##### Genome-Wide Association Study Using Single-Locus Models

Utilizing the transcriptomes of four-year-old roots from 344 core ginseng cultivars, we obtained 39,327 high-quality SNP molecular markers [22]. These SNPs were used for the single-locus model genome-wide association study of Rg1 content BLUP in the population. The TASSEL 5 software Version 5.2.80 [25] was employed for this analysis. Six single-locus model analysis methods were employed, including Generalized Linear Model (GLM), GLM(Q), GLM(PCA), Mixed Linear Model (MLM), MLM(Q + K), and MLM(PCA + K). A Manhattan plot was used to visualize the distribution of *p*-values for each SNP across the entire ginseng genome, while the quantile–quantile (QQ plot) was used to present the correlation results of this model. SNP site selection based on linkage disequilibrium was performed using Plink software Version 1.90, with the following screening criteria: window size: 50, step size: 50, and r^2^ ≥ 0.2. The threshold *p*-value was determined to be 2.54 × 10^−7^. This *p*-value corresponds to a significance threshold (Bonferroni threshold) of −Log10 *p* = 6.59, which was used as the criterion for subsequent selection to identify more accurate loci.

##### Multi-Locus Model Genome-Wide Association Study

In order to enhance the reliability and accuracy of the association analysis, multi-locus association analysis was conducted using the mrMLM software version 4.0.2, wherein significant SNP loci located within a physical distance of ≤20 kb on the same chromosome were summarized as one locus. The software includes five association models: Multi-Locus Random-SNP-Effect Mixed Linear Model (mrMLM) [26], Fast Multi-Locus Random-SNP-Effect Mixed Linear Model (FASTmrMLM) [27], Fast Multi-Locus Random-SNP-Effect Efficient Mixed-Model Association (FASTmrEMMA) [28], Iterative Sure Independence Screening Enhanced EM Bayesian LASSO (ISIS EM-BLASSO) [29], and Polygenic-background-adjusted Least Angle Regression mixed-model Empirical Bayes (pLARmEB) [30]. The kinship matrix was calculated using the TASSEL software Version 5.2.80. Default parameters were used for the GWAS analysis. The threshold for significantly associated SNPs was LOD ≥ 3.0.

##### Determination of Genes within Significantly Associated Intervals in Genome-Wide Association Study

In order to improve the accuracy and reliability of the association analysis, significant loci identified by all six methods of the single-locus model and those present in all five methods of the multi-locus model were selected. Each common significant locus can be regarded as one quantitative trait nucleotide (QTN). Analyzing the 500 Kb interval above and below each QTN locus, the obtained group of candidate genes for Rg1 was designated as Rg1 candidate genes I.

#### 2.2.2. Weighted Gene Co-Expression Network Analysis (WGCNA)

The weighted co-expression network was constructed and modules were identified using the WGCNA package in the R language. Firstly, the expression levels of Rg1 candidate genes I were inputted, and if the expression levels of a candidate gene were zero in over 70% of the cultivars, this gene was not included in WGCNA analysis. Then, sample clustering was computed using the hclust function to identify and remove outlier samples. To meet the requirement of scale-free distribution for network construction, the suitable soft threshold was determined using the pickSoftThreshold() function in the WGCNA package. In this study, a soft threshold (power) value of 8.5 was selected. Subsequently, an unscaled network was constructed using the blockwiseModules function, and adjacency matrices and TOM (topological overlap measure) matrices were generated by calculating the power of gene correlations to form co-expression network modules. To identify hub genes in key module, the top 5 genes with the highest connectivity within key module were selected using Cytoscape 3.9.0 software as core genes in key modules. These core genes were designated as Rg1 candidate genes II.

#### 2.2.3. Study on the Effect of Gene Mutations on Rg1 Content through SNP-Rg1 Content Association Analysis

Among the 4,201,232 SNP/InDel mutation data, all SNPs/InDels of Rg1 candidate genes II were extracted using Vcftools software Version 2.1. The genotypes of 344 cultivars of ginseng were used to analyze the association between candidate genes II SNPs/InDels and Rg1 content, determining the effect of SNP/InDel mutations on Rg1 content. In the SNP/InDel data of Rg1 candidate genes II, the 344 cultivars were grouped according to each SNP genotype, and the difference between the two groups was calculated using an independent sample *t*-test. When *p* ≤ 0.05 was chosen, the genes in Rg1 candidate genes II that were significantly associated with Rg1 content were selected as Rg1 candidate genes III. The impact rate (%) of these significantly associated SNPs on Rg1 content was also calculated. The impact rate (%) was calculated using the following formula: (High Rg1 content—Low Rg1 content)/Low Rg1 content × 100%. The NCBI’s ORF Finder tool was used to analyze whether SNP mutations in Rg1 candidate genes II occurred within open reading frames (ORFs). Based on whether these nucleotide variations caused amino acid changes, it could be determined whether they were synonymous mutations, non-synonymous mutations, or frame-shift mutations in the open reading frame.

#### 2.2.4. Gene Co-Expression Network Analysis

Zhang et al. [31] found that genes with similar functions tend to form a tight interaction network. Therefore, Rg1 candidate genes III was subjected to further screening by performing an interaction network analysis with 15 key enzyme genes involved in ginsenoside biosynthesis. Perl was used to retrieve the expression levels of Rg1 candidate genes III and the 15 key enzyme genes, and the R language was employed for correlation analysis. The analysis results were imported into Biolayout Express3D to generate an interaction network graph. When *p* ≤ 0.05, Rg1 candidate genes III and the 15 key enzyme genes still formed a tight interaction network, confirming these Rg1 candidate genes III as Rg1 candidate genes. As the *p*-value gradually decreased, when *p* ≤ 1.0 × 10^−6^, there were still Rg1 candidate genes forming a tight interaction network with the key enzyme genes involved in ginsenoside biosynthesis, identifying this gene as a key candidate gene for Rg1.

### 2.3. Functional Validation of Key Candidate Genes for Rg1

#### 2.3.1. Validation of MeJA-Induced Regulation

Ginseng adventitious roots grown on solid B5 medium were cut into approximately 3 mm segments and inoculated into 250 mL flasks containing 150 mL of liquid B5 medium supplemented with 0.05% indoleacetic acid (1.2 mL). The inoculation root size was 1.0 g. They were cultured in dark at 22 °C and 110 rpm. On the 25th day of adventitious root liquid culture, 200 μM of MeJA was added for induction treatment, and samples were collected at 0 h, 6 h, 12 h, 24 h, 36 h, 48 h, 60 h, 72 h, 84 h, 96 h, 108 h, and 120 h. Three biological replicates were set up for each treatment group, with adventitious roots not subjected to MeJA induction (0 h) serving as negative controls. At sampling, 2 g of adventitious roots was rapidly frozen in liquid nitrogen and stored at −80 °C for RNA extraction and transcriptome sequencing. The remaining samples were dried in an oven at 35 °C to a constant weight, and their dry weight was measured for ginsenoside extraction and content determination (see Database V).

#### 2.3.2. Validation with RNA Interference (RNAi)

RNAi genetic transformation technology is one of the most commonly used techniques to verify the function of candidate genes. There have been reports on verifying the function of key enzyme genes in ginsenoside biosynthesis through RNAi technology. This study also used RNAi technology to verify the role of Rg1 key candidate gene in Rg1 biosynthesis. The reverse fragment (100 bp) of the Rg1 key candidate gene was seamlessly cloned between the *Bam*HI and *Xba* I restriction sites of the pFGC5941 plasmid, and then the forward fragment (100 bp) of the gene was connected between the *Asc* I and *Swa* I restriction sites of the pFGC5941 plasmid containing the reverse fragment to construct a recombinant plasmid. The recombinant plasmid was transformed into *Agrobacterium rhizogenes* C58C1 competent cells and screened on YEP solid medium containing rifampicin (50 ng/µL), kanamycin (50 ng/µL), and streptomycin (50 ng/µL) to obtain positive strains. These strains were cultured in YEP liquid medium with the same antibiotics until OD600 = 0.4. The bacteria were collected by centrifugation and resuspended in 1/2 MS liquid medium containing acetosyringone (AS) to OD600 = 0.5. Pre-cultured ginseng adventitious roots were infected with bacterial suspension for about 15 min. After infection, the roots were cultured on solid 1/2 MS medium with 20 µM of AS for 48 h in dark at 22 °C to 25 °C, then transferred to 1/2 MS medium with cefotaxime (100 ng/µL) until roots grew. Each root was cultured in liquid 1/2 MS medium (22 °C, 110 rpm) for proliferation and subculture to establish single root lines. Some single root lines were frozen in liquid nitrogen and stored at −80 °C for total RNA extraction and real-time quantitative PCR (qRT-PCR) analysis. Others were dried in an oven at 35 °C to constant weight and stored at 4 °C for ginsenoside extraction and content detection.

### 2.4. Exploration of the Molecular Mechanism of the PgRg1-3 Gene in Rg1 Biosynthesis

#### 2.4.1. Functional Annotation of the *PgRg1-3* Gene by GO and KEGG Pathway Localization, and Homology Analysis with Ginseng *CYP* Genes

Blast2GO v.6.0.3 was used for functional annotation of the *PgRg1-3* gene by GO and KEGG pathway localization. The nucleotide sequence of the *PgRg1-3* gene was compared with the nucleotide sequences of three *CYP* genes (*CYP716A53v2_1*, *CYP716A52v2_3*, *CYP716A47_1*) controlling ginsenoside biosynthesis in ginseng, using BLAST to analyze their homology, determining whether the *PgRg1-3* gene is a new *CYP* gene in ginseng.

#### 2.4.2. Gene Expression Patterns at Different Times and in Different Cultivars

Zhang et al. [31] found that different transcripts of the same gene have different biological functions. Therefore, this study investigated the gene expression patterns at different times and in different cultivars using transcripts of *PgRg1-3* and 11 key enzyme genes involved in ginsenoside biosynthesis. The expression levels of the *PgRg1-3* gene and the 15 transcripts of key enzyme genes for ginsenoside biosynthesis were extracted from Databases III, IV, and I, and heatmaps were constructed to explore the spatiotemporal expressions of *PgRg1-3* gene and key enzyme gene transcripts for ginsenoside biosynthesis and whether their expressions are similar.

#### 2.4.3. The Impacts of Gene Co-Expression Network Variations on Rg1 Biosynthesis

The expression levels of *PgRg1-3* gene and 15 transcripts of key enzyme genes for ginsenoside biosynthesis were extracted. Based on the Rg1 content of 42 ginseng cultivars in the core germplasm population, they were divided into three groups: low-Rg1-content group (Low), medium-Rg1-content group (Mid), and high-Rg1-content group (High), with 14 cultivars in each group. The expression correlations of these three groups of genes were calculated using the R software package Version 4.1.2, and the results were imported into Biolayout Express3D separately to construct gene co-expression networks. The impacts of the number and types of genes (nodes) and gene interactions (edges) in the co-expression network on Rg1 ginsenoside biosynthesis were analyzed.

#### 2.4.4. Genetic Effects of SNP Mutations in the *PgRg1-3* Gene on Rg1 Biosynthesis

SNPs in the *PgRg1-3* gene have three genotypes in 344 ginseng cultivars: two homozygous types, AA and GG, and one heterozygous type, AG. When calculating the genetic effects, it is first necessary to determine which of the two homozygous types is the favorable allele and which is the unfavorable allele. If the Rg1 content of the homozygous AA group is higher than that of the homozygous GG group, “A” is the favorable allele, and “G” is the unfavorable allele. Upon determining the favorable allele, the genetic effects of the heterozygous AG on Rg1 content are analyzed. AG > AA is an over-dominant effect (OD); AG = AA is a complete dominant effect (CD); (AA + GG)/2 < AG < AA is an incomplete dominant effect (ID); AG = (AA + GG)/2 is an additive effect (AD); GG < AG < (AA + GG)/2 is a negative incomplete dominant effect (NID); AG = GG is a negative complete dominant effect (NCD); AG < GG is a negative over-dominant effect (NOD).

## 3. Results

### 3.1. Identification of Candidate Genes and Key Candidate Genes for Ginsenoside Rg1 Biosynthesis

#### 3.1.1. Genome-Wide Association Study

##### Statistical Analysis of Ginsenoside Rg1 Content

Statistical analysis of the Rg1 content in the roots of 4-year-old plants of 344 cultivars in ginseng core germplasm resources showed that the Rg1 content ranged from 0.12 mg/g to 1.681 mg/g, with an average content of 0.632 mg/g, a standard deviation of 31.24%, and a coefficient of variation of 55.77%. The results indicate that there is a large variation in Rg1 content among different cultivars. To reduce the influence of environmental factors on Rg1 content and improve the accuracy of complex quantitative traits, the BLUP method was used to calculate the Rg1 content in each sample, and the Rg1 content was normally distributed (Supplementary Figure S1). These results indicate that the variation in Rg1 content is relatively uniform in the core germplasm resources, and this trait can be used for GWAS analysis.

##### Genome-Wide Association Study of Ginsenoside Rg1 Content Using Single-Locus Models

**Figure 1:**
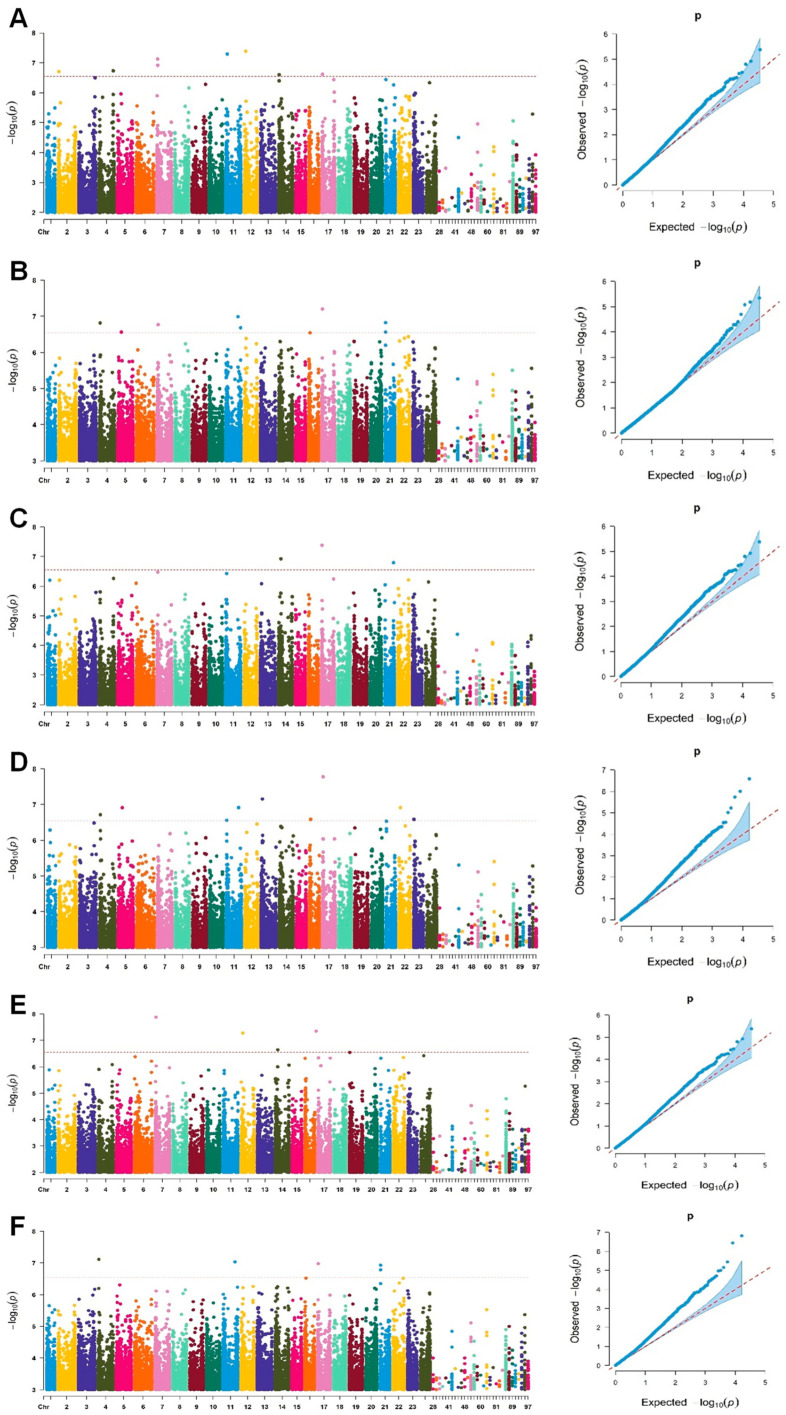
Manhattan plots of –Log10(*p*) vs. chromosomal position of SNP markers associated with ginsenoside Rg1 content and quantile–quantile (QQ) plots in a Jilin ginseng core collection using six single-locus models, (**A**) GLM, (**B**) GLM(Q), (**C**) GLM(PCA), (**D**) MLM(K), (**E**) MLM(Q + K), and (**F**) MLM(PCA + K). A red horizontal dashed line indicates the significant threshold –Log10(*p*) = 2.54 × 10^−7^ from Bonferroni correction method. The *x*-axis shows chromosomes and contigs.

Using six single-locus association models, including GLM, GLM(Q), GLM(PCA), MLM(K), MLM(K + Q), and MLM(K + PCA) in TASSEL 5 software, a genome-wide association study was conducted using Rg1 content as the phenotype data (Figure 1, Supplementary Table S1). A total of 37 SNPs significantly associated with Rg1 content were detected, distributed across 14 chromosomes, explaining phenotypic variation rates between 4.1% and 24.5%. Among them, 15 loci were redundant, resulting in 22 significant associated loci being obtained.

##### Genome-Wide Association Study of Ginsenoside Rg1 Content Using Multi-Locus Models

Using five multi-locus association models, including mrMLM, FASTmrMLM, FASTmrEMMA, ISIS EM-BLASSO, and pKWmEB in the mrMLM 4.0 software package, a genome-wide association study was conducted using Rg1 content as the phenotype data (Supplementary Figure S2, Supplementary Table S1). A total of 67 SNPs significantly associated with Rg1 content were detected, distributed across 21 chromosomes and 2 contigs, explaining 0.78% to 9.29% of phenotypic variation. Among them, 17 loci were redundant, resulting in 50 significant associated loci being obtained.

##### Identification of Genes in Significantly Associated Regions through Genome-Wide Association Study

**Figure 2:**
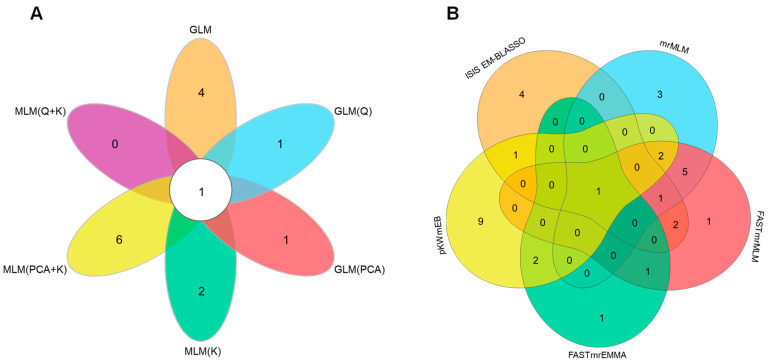
Two QTNs were consistently identified, with one by all six single-locus models (**A**) and the other by all five multiple-locus models (**B**).

Using six single-locus models and five multi-locus models, a total of 69 loci significantly associated with Rg1 content were identified. Among them, one significant associated locus (QTN) was detected by all six single-locus models (Figure 2A), and one significant associated locus (QTN) was detected by all five multi-locus models (Figure 2B). These two QTNs are located at 5,187,718 bp on chromosome 17 and 29,713,841 bp on chromosome 3 (Supplementary Figure S3). Sequences upstream and downstream of each QTN within 500 kb were extracted, resulting in a total of 541 genes as candidate genes I for Rg1 biosynthesis (Supplementary Table S2).

#### 3.1.2. WGCNA Analysis

**Figure 3:**
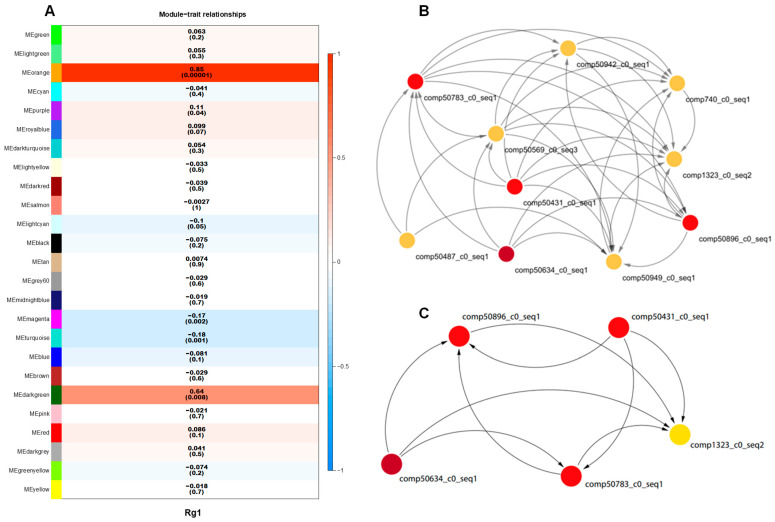
Module–trait association, orange module gene expression network, and identification of hub genes. (**A**) Module–trait association. Each row corresponds to a module gene and the column to Rg1 content. The numbers of each row indicate correlation coefficient (*r*) in the top and the *p*-value in parenthesis. (**B**) Network of Rg1 biosynthesis candidate genes with the highest *r* = 0.85 in orange module. The nodes show genes and the edges show the interactions between two genes. (**C**) Genes with top-5 degree were considered as hub genes.

The expression levels of 541 candidate genes for Rg1 biosynthesis were extracted using Perl, and genes with zero expression in over 70% of samples were removed, leaving 410 candidate genes for Rg1 biosynthesis to undergo hierarchical clustering based on expression levels. When power = 0.85, a total of 26 expression modules were identified (Supplementary Figure S4A,B). The number of genes within these modules ranged from 5 to 26, with the dark gray module containing the most genes (26) and the yellow and salmon modules containing the fewest (5) (Supplementary Table S3). Correlation analysis between these modules and Rg1 content revealed that 10 modules were positively correlated with Rg1 content, while 15 modules were negatively correlated. Among them, the orange module had a correlation coefficient of 0.85, with *p* = 1.0 × 10^−5^ (Figure 3A), indicating it as a key module. A weighted co-expression network was constructed using the 10 genes from the key module (Figure 3B), and genes with connectivity weight >0.4 in the network were further screened to identify the top five genes by connectivity, designated as hub genes within the orange module (Figure 3C). These five hub genes were designated as candidate genes II for Rg1 biosynthesis.

#### 3.1.3. Association Analysis of SNPs with Rg1 Content Investigating the Impact of Gene Mutations on Rg1 Content

**Figure 4:**
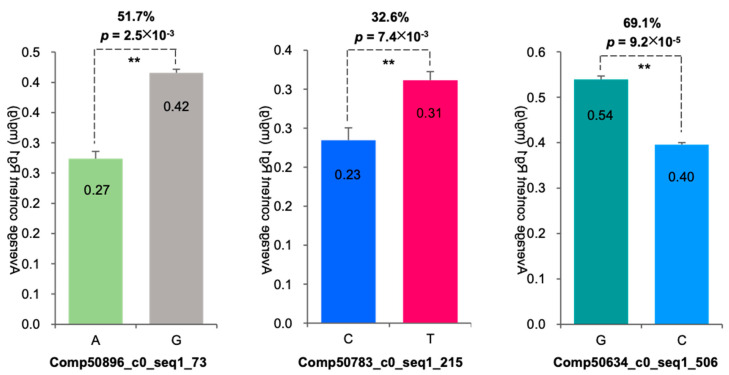
Impacts of the SNP mutations of three Rg1 candidate genes on Rg1 contents in roots of four-year-old plants of 344 cultivars in the ginseng core collection. The mutation of each candidate gene is shown as the name and the position of the mutation. The impact of each mutation is shown as a percentage of the *p*-value (** for a two-tailed significance of *p* < 0.01). For details, see Supplementary Table S4.

Among the 326 SNPs identified in the five candidate genes II for Rg1 biosynthesis, association analysis with Rg1 content revealed that 57 SNPs were significantly correlated with Rg1 content (*p* ≤ 0.05) within the three candidate genes II, accounting for 17.5% of all SNPs. Among these, 50 SNPs were located within the ORF, and 7 were outside the ORF. Of the 50 SNPs within the ORF, 37 (74%) were non-synonymous mutations, 7 (14%) were synonymous mutations, and 6 (12%) were ORF shift mutations. The impact of each SNP on Rg1 content ranged from 16.67% to 81.38% (Figure 4, Supplementary Table S4). These three candidate genes II for Rg1 biosynthesis were designated as candidate genes III for Rg1 biosynthesis.

#### 3.1.4. Network Analysis of Candidate Genes III for Rg1 Biosynthesis and 15 Key Enzyme Gene Transcripts Involved in Ginsenoside Biosynthesis

**Figure 5:**
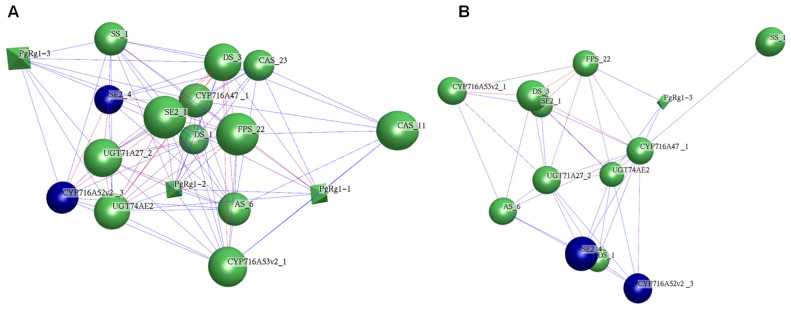
Co-expression network of three Rg1 candidate genes and 15 key enzyme gene transcripts involved in ginsenoside biosynthesis in the four-year-old roots of 344 cultivars of the ginseng core collection. (**A**) Co-expression network of 18 genes was constructed at *p* ≤ 0.05. The rhombs (nodes) represent Rg1 candidate genes, the balls (nodes) represent key enzyme genes for ginsenoside biosynthesis, and the lines (edges) represent interactions between genes. The network consists of all three Rg1 candidate genes and 14 ginsenoside biosynthesis genes except *ß-AS_1*, and two clusters are shown by different colors. (**B**) At *p* ≤ 1 × 10^−6^, only the *PgRg1-3* was in the co-expression network with key enzyme genes involved in ginsenoside biosynthesis, thus this gene is named the Rg1 key candidate gene for validation of its biological function.

The co-expression network of Rg1 candidate genes III and 15 ginsenoside biosynthesis key enzyme gene transcripts was constructed based on their expression levels in 344 cultivars. At *p* ≤ 0.05, a network with 18 nodes and 99 edges was formed (Figure 5A). This indicates that Rg1 candidate genes III shares functional similarity with ginsenoside biosynthesis key enzyme gene transcripts and may be involved in Rg1 biosynthesis. These three candidate genes III were finally identified as Rg1 candidate genes and named *PgRg1-1*, *PgRg1-2*, and *PgRg1-3*. At *p* ≤ 1 × 10^−6^, the candidate gene *PgRg1-3* remained in the same interaction network as the ginsenoside key enzyme gene transcripts, indicating a closer relationship between *PgRg1-3* and ginsenoside biosynthesis genes. Therefore, *PgRg1-3* is designated as a key candidate gene for Rg1 biosynthesis (Figure 5B).

### 3.2. Functional Validation of the PgRg1-3 Key Candidate Gene for Rg1 Biosynthesis

#### 3.2.1. MeJA-Induced Regulation 

**Figure 6:**
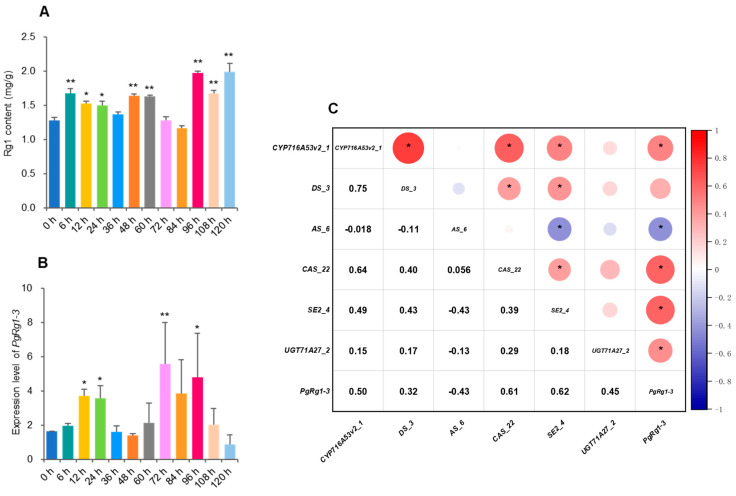
Ginseng adventitious roots treated with MeJA from 6 h to 120 h, relative to the control roots not treated with MeJA (0 h). (**A**) Rg1 content variations in the adventitious roots treated with MeJA, relative to the control roots. (**B**) The expression variation of *PgRg1-3* candidate gene in the adventitious roots treated with MeJA, relative to the control roots. (**C**) Correlation of *PgRg1-3* candidate gene and key enzyme genes involved in ginsenoside biosynthesis after MeJA induced ginseng adventitious roots. The “*” and “**” asterisks indicate the difference between MeJA treated and control roots is significant at *p* ≤ 0.05 and 0.01, respectively. The remaining MeJA treated roots not labelled with asterisk are not significantly different from the control roots.

The MeJA-induced regulation of Rg1 biosynthesis of ginseng adventitious roots at different time periods was investigated by high-performance liquid chromatography (HPLC), showing significant changes in Rg1 content compared to the control at 6 h, 12 h, 24 h, 48 h, 60 h, 96 h, 108 h, and 120 h (*p* ≤ 0.05, 0.01) (Figure 6A). This indicates that MeJA regulates Rg1 biosynthesis in ginseng adventitious roots. Concurrently, the expression levels of *PgRg1-3* key candidate gene and ginsenoside biosynthesis key enzyme genes (*CYP716A53v2_1*, *DS_3*, *ßAS_6*, *CAS_22*, *SE2_4*, *UGT71A27_2*) were analyzed. It was found that the expression levels of *PgRg1-3* key candidate genes were significantly altered compared to the control at 12 h, 24 h, 72 h, and 96 h of MeJA treatment (Figure 6B), and the expressions levels of ginsenoside key enzyme gene also varied significantly at certain induction time points (Supplementary Figure S5), indicating that the expressions of *PgRg1-3* key candidate gene and key enzyme genes are not only regulated by MeJA but also affect the significant changes in Rg1 content, confirming the involvement of *PgRg1-3* in Rg1 biosynthesis. To further demonstrate the involvement of *PgRg1-3* in Rg1 biosynthesis, correlation analysis between *PgRg1-3* and key enzyme genes was conducted. It was found that the expression of *PgRg1-3* was significantly correlated with the expressions of *CYP716A53v2_1*, *ßAS_6*, *CAS_22*, *SE2_4*, and *UGT71A27_2* (Figure 6C), further confirming the involvement of *PgRg1-3* key candidate gene in Rg1 biosynthesis.

#### 3.2.2. Genetic Transformation RNA Interference (RNAi) 

**Figure 7:**
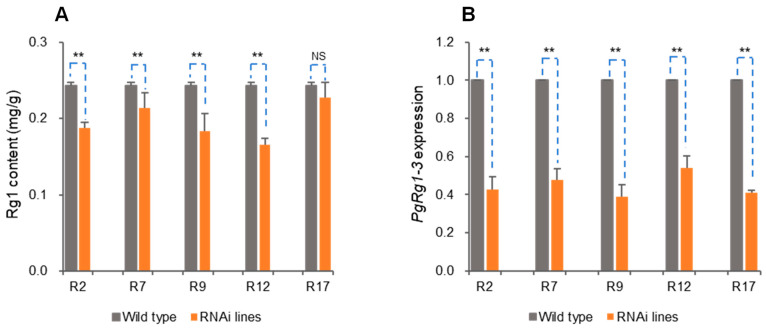
Functional validation of *PgRg1-3* in Rg1 biosynthesis with RNAi genetic transformation. (**A**) Rg1 contents in transgenic lines and WT control determined by HPLC. (**B**) Expression of *PgRg1-3* in transgenic lines and WT control determined by qPCR. The difference in Rg1 content or *PgRg1-3* expression between transgenic lines and WT control was conducted by *t*-test, with “**” for significance at *p* ≤ 0.01 and “NS” for non-significance.

RNAi technology was used to further verify the function of *PgRg1-3* key candidate gene. Ginseng adventitious roots were genetically transformed with RNAi targeting *PgRg1-3* (Supplementary Figure S6), resulting in 62 positive hairy root lines. Among these, five stable positive hairy root lines were chosen for measuring Rg1 content and *PgRg1-3* gene expression, with ginseng hairy roots transformed by the original strain of *A. rhizogenes* C58C1 serving as a negative control. In these five positive hairy root lines, the Rg1 content in four of them significantly decreased compared to the control, with reductions of 25.16%, 12.44%, 24.82%, and 31.95%, respectively (Figure 7A), indicating the control of Rg1 biosynthesis by *PgRg1-3*. Meanwhile, the expression levels of the *PgRg1-3* gene significantly decreased compared to the control, with reductions ranging from 1.79 to 2.36 times (Figure 7B), indicating that the expression of this gene was interfered at different levels, affecting Rg1 biosynthesis.

### 3.3. Exploration of the Molecular Mechanism of the PgRg1-3 Gene in Rg1 Biosynthesis 

#### 3.3.1. Functional Annotation of *PgRg1-3* Gene by GO, KEGG Localization, and Homology Comparison with Ginseng *CYP* Genes

The *PgRg1-3* gene is a cytochrome P450 gene (*PgCYP*), with a cDNA length of 1280 bp and a full length of 1513 bp on the genome, containing two exons and one intron. The GO functional annotation of the *PgRg1-3* gene is oriented towards cellular component (CC) and molecular function (MF). At level 2, it is involved in membrane composition (integral component of membrane, GO:0016021), monooxygenase activity (monooxygenase activity, GO:0102375), and oxidoreductase activity (oxidoreductase activity, GO:0020037). The KEGG pathway annotation includes the fatty acid degradation pathway (Supplementary Figure S7A). The nucleotide sequence of the *PgRg1-3* gene was compared with that of the cloned *CYP716A53v2_1*, *CYP716A52v2_3*, and *CYP716A47_1* genes in ginseng, revealing only about 40% similarity, indicating that the *PgRg1-3* gene is a new cytochrome P450 gene involved in Rg1 biosynthesis in ginseng (Supplementary Figure S7B).

#### 3.3.2. Gene Expression Patterns at Different Spatiotemporal Points and in Different Cultivars

The expression of the *PgRg1-3* gene and 15 ginsenoside synthesis key enzyme gene transcripts was studied in roots of ginseng aged 5, 12, 18, and 25 years. It was found that the expression level of *PgRg1-3* in 25-year-old ginseng roots was 2.82 times higher than that in 5-year-old ginseng roots, and with the increase in ginseng age, the gene expression showed an increasing trend. Among the 15 key enzyme gene transcripts involved in ginsenoside biosynthesis, some were expressed the most in 5-year-old roots, such as *ß-AS_1*, *SE2_1*, *DS_3*, *CYP716A52v2_3*, *FPS_22*, and *UGT74AE2,* while others were expressed the most in 25-year-old roots, such as *ß-AS_6*, *CAS_23*, *SS_1*, *SE2_4*, *CYP716A53v2_1*, and *DS_1*. *PgRg1-3* clustered with the *CAS_23* gene (Supplementary Figure S8A), indicating their similar expression and similar regulatory role in Rg1 biosynthesis. These results indicate that the expression levels of the same gene at different growth and development stages are different, and they interact with the expression of other genes to control the biosynthesis of ginsenosides at different developmental stages.

The expression of *PgRg1-3* and 15 key enzyme gene transcripts involved in ginsenoside biosynthesis was studied in 14 different tissues of ginseng. It was found that the expression level of *PgRg1-3* was highest in the fiber root, followed by leg roots, arm roots, and stems, and lowest in the root epiderm and root heart of the main root. Moreover, no regularity was found between *PgRg1-3* and other ginsenoside biosynthesis key enzyme genes (Supplementary Figure S8B), suggesting that this gene may have the greatest impact on Rg1 ginsenoside biosynthesis in fiber roots.

The expression of the *PgRg1-3* gene and 15 key enzyme gene transcripts involved in ginsenoside biosynthesis in the 4-year-old roots of 42 cultivars is shown in Supplementary Figure S8C. The expression of *PgRg1-3* was extremely high in cultivar S22, with significant variation in expression among other cultivars, without any obvious regularity. Most of the key enzyme genes involved in ginsenoside biosynthesis, except *CAS_11* and *β-AS_1*, showed higher expression in the roots of cultivar S23. *PgRg1-3* clustered with *UGT74AE2*, indicating their similar expression and similar regulatory role in Rg1 biosynthesis. These results indicate that the difference in Rg1 content in roots of different cultivars may be related to the different expression activities of these genes in roots of different cultivars.

#### 3.3.3. Impact of the Changes in Gene Co-Expression Networks on Rg1 Biosynthesis

**Figure 8:**
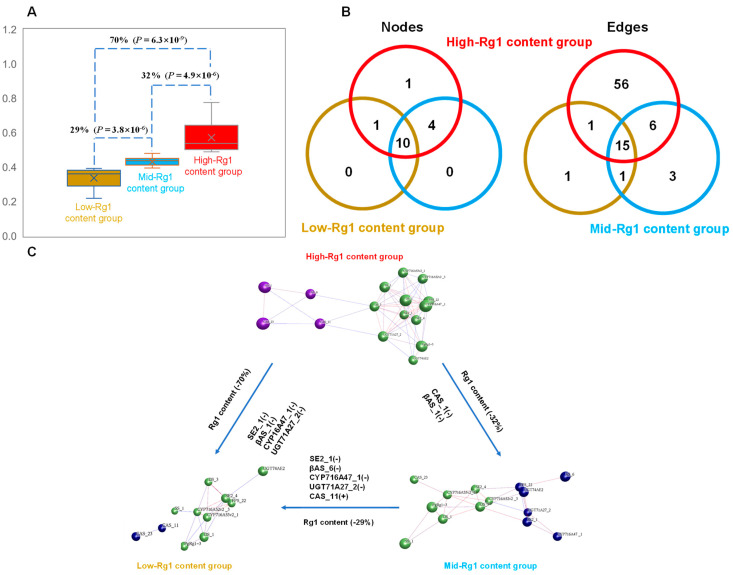
Effect of co-expression network variation of *PgRg1-3* and 15 key enzyme gene transcripts for ginsenoside biosynthesis on Rg1 content among cultivar groups with high-, mid-, and low-Rg1 content. (**A**) Variation in Rg1 content among cultivar groups. The percentage represents Rg1 content variation between two groups and the *p*-value represents significant differences in Rg1 content between two groups. (**B**) Effect of variation of the gene nodes and the gene interaction edges in the network on Rg1 content. High-Rg1-content group is shown by red color, mid-Rg1-content group by brown color, and low-Rg1-content group by blue color. (**C**) Effect of number and type of genes and edges among the networks in high-, mid-, and low-Rg1-content groups.

Based on the high and low levels of Rg1 content, the 42 cultivars were divided into the high-Rg1-content group, medium-Rg1-content group, and low-Rg1-content group. Through *t*-test analysis, it was found that the difference in Rg1 content between each group ranged from 29% to 70%, with significant differences (*p* ≤ 4.9 × 10^−6^) (Figure 8A). Is this significant difference related to the co-expression of genes controlling Rg1 biosynthesis? In this study, co-expression network analysis of *PgRg1-3* and 15 key enzyme gene transcripts involved in ginsenoside biosynthesis was conducted for these three groups, revealing that the number of genes (nodes) in the high, medium, and low groups was 16, 14, and 11, respectively (Figure 8B), and the types of genes in these three groups were also different (Figure 8C). For example, there was no *CAS_11* gene in the medium group, while there was a *CAS_11* gene in the low group. These results indicate that the number and types of genes affect Rg1 biosynthesis. In terms of gene interactions (edges), the high group had the most gene interactions, with 78, followed by the medium group with 25 and the low group with the fewest at 18 (Figure 8B). This indicates that the interaction between genes also affects Rg1 biosynthesis. Due to the different numbers and types of genes in each group, the types of interactions between genes are also different (Figure 8C) and affect Rg1 biosynthesis. These results indicate that not only does the co-expression network of the *PgRg1-3* gene and key enzyme genes control Rg1 biosynthesis, but also, the changes in their interaction relationships also affect Rg1 biosynthesis.

#### 3.3.4. Analysis of the Impact and Genetic Effects of SNP Mutations in the *PgRg1-3* Gene on Rg1 Biosynthesis

**Figure 9:**
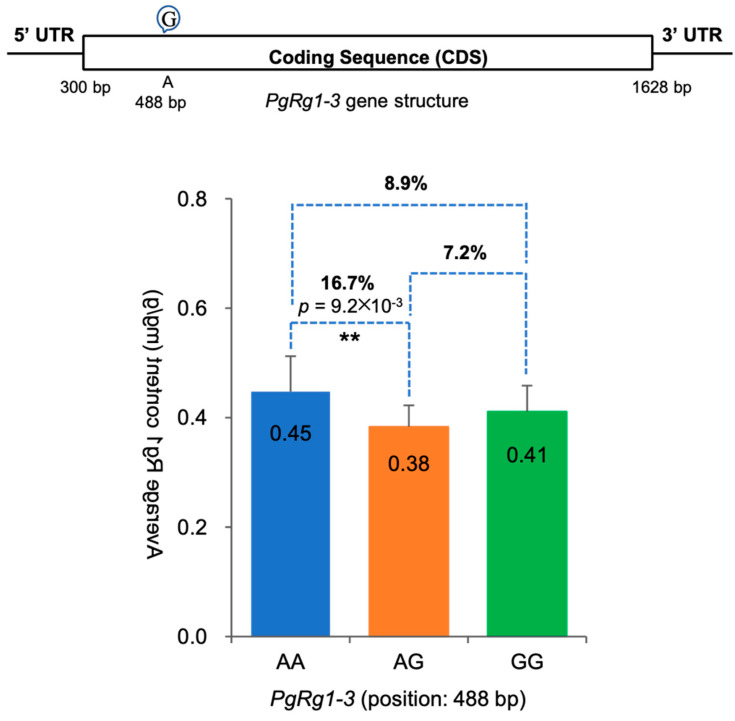
The structure of the *PgRg1-3* gene and the negative over-dominant effect of its SNP mutation at position 488 bp on Rg1 content in the 344 cultivars of the Jilin ginseng core collection. The genotypes of SNP mutations are shown in the x-axis. The number in the bar presents Rg1 content mean in a genotype and the percentage shows the impact of SNP mutation on Rg1 content. “**” for significance level of *p* ≤ 0.01.

A total of 17 SNPs were identified in the *PgRg1-3* gene, among which 12 SNP mutations significantly affected the Rg1 content, with the impact ranging from 16.7% to 80.3% (Supplementary Table S4). Among the 17 SNPs, only one SNP exhibited three genotypes (AA, AG, GG), allowing for genetic effect analysis. This mutation site is located at position 488 bp, with A mutating to G, resulting in an amino acid change from aspartic acid to serine; this mutation is a nonsynonymous mutation. Analysis of Rg1 content in two homozygous types showed that the Rg1 content of the AA genotype was higher than that of the GG genotype, indicating that A is the favorable allele and G is the unfavorable allele. The Rg1 content of the AG heterozygous type was significantly lower than that of the AA genotype (*p* ≤ 9.2 × 10^−3^), decreasing by 16.7% (Figure 9), indicating that the *PgRg1-3* gene exhibits a negative over-dominant genetic effect on Rg1 biosynthesis.

## 4. Discussion

Most biological traits and metabolic pathways are controlled by multiple genes. Accurately and efficiently cloning genes that control these traits and processes at the whole-genome level is a significant research topic. The premise of gene cloning is to first identify candidate genes that control the target traits and then verify their functions through methods such as genetic transformation or gene editing. Current methods for identifying candidate genes at the whole-genome level include QTL mapping, eQTL, and GWAS. However, these methods have limitations. In QTL mapping, multiple genes may be present in one QTL or genomic interval, making it difficult to pinpoint the exact genes controlling the target traits. Furthermore, QTL mapping is constrained by factors such as the availability of mapping populations, high-density genetic maps, and genomic research foundations. Because most biological traits are quantitative and regulated by multiple genes, many of these genes have low copy numbers, complicating the identification process. GWAS addresses some of these limitations by directly locating genes associated with multiple traits using natural populations or germplasm resources. However, GWAS also identifies many genes within the mapping interval, necessitating additional methods to identify the candidate genes controlling the target traits [32].

Ginseng is a perennial, cross-pollinating plant with a large and complex genome. Compared to field crops such as maize, rice, and soybean, research on its genome and genetic breeding lags behind. Ginseng lacks artificial hybridization populations and genetic maps, making it impossible to use methods such as QTL mapping to identify new candidate genes in ginseng. Currently, the identification of genes related to ginsenoside biosynthesis mainly relies on the sequences of key enzyme genes in the triterpenoid metabolic pathways of known species, identifying their homologous sequences in ginseng as candidate genes. This method is inefficient and cannot identify all or most of the genes that control a specific ginsenoside, nor can it directly identify new genes that control ginsenosides in ginseng.

In order to accurately and efficiently identify candidate genes controlling ginsenoside biosynthesis at the whole-genome level, this study integrated four candidate gene identification methods to gradually and comprehensively identify candidate genes for ginsenoside Rg1 biosynthesis in ginseng. These methods include GWAS (*p* < 1.0 × 10^−6^), WGCNA (*p* < 1.0 × 10^−5^), SNP-Rg1 content correlation analysis (*p* < 5.0 × 10^−2^), and gene co-expression network analysis (*p* < 5.0 × 10^−2^). The cumulative *p*-value of this method is less than 5.0 × 10^−15^, indicating an extremely stringent threshold, ensuring the accuracy and rigor of candidate gene identification. Additionally, using a stepwise analysis approach allows for the maximal identification of most candidate genes controlling the target traits. Furthermore, this method is based on different analytical principles, one being linkage disequilibrium (LD) and the other being the correlation between gene expression and traits. Using two analytical principles is more accurate than using one.

MeJA is an important stress signal and a commonly used inducer in plant tissue culture [33,34]. By adding MeJA, the JA signaling pathway within plants can be activated, triggering stress responses to enhance the accumulation of secondary metabolites. Adding MeJA to the cultures of various plants in the Araliaceae family, such as ginseng, *Panax quinquefolius*, and *Panax notoginseng*, leads to changes in the content of ginsenosides and the expressions of genes related to ginsenoside biosynthesis to explore the ginsenoside biosynthesis pathway. Currently, key enzyme genes involved in ginsenoside biosynthesis in ginseng, such as *PgSS* [35], *PgSE* [36], *PgMVD* [37], *PgFPS* [38], *PgDDS* [39], *PgUGT* [40], *PgCYP450* [41], and *PgRb1* [24], are all regulated by MeJA, confirming their involvement in ginsenoside biosynthesis. The transcription factor *PgMYB2* [42] in ginseng was also identified using this method. Therefore, MeJA-induced regulation has been widely used to verify the biological functions of candidate genes for ginsenoside biosynthesis in ginseng.

In this study, the biological function of the *PgRg1-3* candidate gene was verified using MeJA exogenous regulation methods. The results showed that the Rg1 content in ginseng adventitious roots induced at different times significantly changed compared to the control, indicating that Rg1 biosynthesis is regulated by MeJA. Additionally, the expression levels of the *PgRg1-3* candidate gene and key enzyme genes involved in ginsenoside biosynthesis in ginseng also significantly changed compared to the control. Furthermore, there was a significant correlation in the expression of the *PgRg1-3* candidate gene and key enzyme genes involved in ginsenoside biosynthesis, indicating that the *PgRg1-3* candidate gene is involved in the Rg1 biosynthesis. Therefore, this study successfully verified the involvement of the *PgRg1-3* candidate gene in Rg1 biosynthesis using MeJA exogenous regulation.

RNAi genetic transformation technology is a commonly used technique to validate the biological functions of candidate genes. In ginseng, the genes with known biological functions validated using RNAi technology include *PgSE1* [43], *PgDDS* [44], *CYP716A47* [45], *UGT94Q2* [46], and *CYP716A53v2* [47]. In this study, RNAi technology was used to validate the biological function of the *PgRg1-3* candidate gene. RNAi adventitious roots of *PgRg1-3* candidate genes were obtained through *A. rhizogenesis*-mediated transformation, resulting in a significant decrease in the expression of this candidate gene and the Rg1 content. The RNAi genetic transformation results confirmed that the *PgRg1-3* candidate gene controls Rg1 biosynthesis and exhibits positive regulation of Rg1 biosynthesis. This study utilized two methods, MeJA exogenous regulation and RNAi, to validate the control of Rg1 biosynthesis by the *PgRg1-3* candidate gene, leading to successful cloning of the gene controlling Rg1 biosynthesis and supporting the accuracy of the candidate gene identification method used in this study.

Research has shown that the content of ginsenosides increases with the age of ginseng. This study found that the expression level of the *PgRg1-3* gene also tends to increase in older ginseng roots, indicating that the expression pattern of the *PgRg1-3* gene is similar to the accumulation pattern of ginsenosides, further demonstrating the important role of the *PgRg1-3* gene in Rg1 biosynthesis. Analysis of the expression patterns of the *PgRg1-3* gene in 14 tissues of 4-year-old ginseng revealed that *PgRg1-3* is most highly expressed in the fiber roots, possibly promoting the biosynthesis of Rg1 in the fiber roots. There are significant differences in the expression patterns of the *PgRg1-3* gene in 4-year-old roots of different cultivars. In the cultivar S22, the expression of the *PgRg1-3* gene is particularly prominent. These results indicate that differences in gene expression lead to differences in Rg1 content in different cultivars.

In the study on the impact of gene interaction network changes on Rg1 biosynthesis, it was found that the content of Rg1, as a quantitative trait, is not only controlled by multiple genes but also regulated by gene interaction networks. This finding is consistent with the results of Zhang et al. [31]. The impact on Rg1 biosynthesis involves not only the number and type of genes in the interaction network but also the quantity, types, and tightness of gene interactions. This research further demonstrates the complexity of Rg1 biosynthesis. Studying only one or two genes makes it difficult to reveal the molecular mechanisms of Rg1 biosynthesis. Moreover, in genetic improvement for ginseng quality, altering one or two genes may not necessarily increase the Rg1 content. Therefore, accurately and efficiently cloning ginsenoside genes at the whole-genome level is not only crucial for studying the mechanism of ginsenoside biosynthesis but also plays a critical role in efficient ginseng breeding.

Heterosis refers to the phenomenon where the offspring of hybrids are superior to their parents in one or more traits, or where the traits of heterozygotes are superior to those of homozygotes. For instance, hybrids or heterozygotes may exceed their parents or homozygotes in traits such as vigor, reproductive capacity, yield, secondary metabolite production, and stress tolerance, making them widely applicable in agricultural production. Molecular mechanism of heterosis is a key theoretical basis for improving breeding efficiency, especially for hybrid breeding. Currently, the study of heterosis often employs QTL mapping followed by genetic effect analysis. However, QTL mapping often involves multiple genes within the QTL interval, possibly hundreds of genes, making it difficult to identify specific gene controlling the heterotic traits. This greatly limits the elucidation of the genetic mechanism of heterosis. Genes are the core elements for revealing biological traits, metabolic pathways, heterosis, and other life phenomena. However, so far, very few genes controlling heterosis have been cloned, with only one dominant heterosis gene controlling fruit yield cloned in tomatoes, known as *SINGLE FLOWER TRUSS (SFT)* [48]. This study cloned a gene controlling Rg1 biosynthesis, *PgRg1-3*, which is a negative dominant gene. Although it has no practical value in production, it represents one form of heterosis and can be used to study the molecular mechanism underlying the formation of heterosis.

## 5. Conclusions

This study integrated GWAS, WGCNA, genic SNP-content association analysis, and gene co-expression network analysis to identify candidate genes for Rg1 biosynthesis. Three candidate genes for Rg1 and one key candidate gene, *PgRg1-3*, were identified. A total of 73 SNPs significantly associated with Rg1 content were also discovered. The biological function of *PgRg1-3* in Rg1 biosynthesis was validated through MeJA regulation and RNAi technology, culminating in the successful cloning of a gene controlling Rg1 biosynthesis. This study investigated the temporal and spatial expressions of *PgRg1-3*, as well as the impacts of gene co-expression network dynamics and heterosis on Rg1 biosynthesis. These findings provide genetic resources and a theoretical foundation for ginseng genetic engineering and gene breeding. Effective SNP molecular markers identified in this study contribute to ginseng variety evaluation and genome selection. Moreover, this study offers insights and methodologies for identifying and cloning other genes related to ginsenoside biosynthesis.

## Data Availability

The datasets generated during and analyzed during the current study and all plant materials are available from the corresponding author on reasonable request.

## Supplementary Materials

The following supporting information can be downloaded at https://www.mdpi.com/article/10.3390/plants13131784/s1: Figure S1: Distribution of Rg1 content BLUP in four-year-old ginseng plant roots among 344 cultivars in a ginseng core collection representing the genetic variation and diversity of Jilin ginseng; Figure S2: Manhattan plots of –Log10(*p*) vs. chromosomal position of SNP markers associated with ginsenoside Rg1 content and quantile–quantile (QQ) plots in a Jilin ginseng core collection using five multiple-locus models, (A) mrMLM, (B) FASTmrMLM, (C) FASTmrEMMA, (D) pKWmEB, and (E) ISIS EM-BLASSO. A blue dashed line represents the significant –Log10(*p*) = 2.54 × 10^−7^ from the Bonferroni correction method; Figure S3: LD plot showing a 500 kb QTN region LD pattern among the 61 SNPs genotyped; Figure S4: Module detection of weighted gene co-expression network; Figure S5: Ginseng adventitious roots treated with MeJA from 6 h to 120 h, relative to the control roots not treated with MeJA (0 h); Figure S6: Genetic transformation of the *PgRg1-3* gene into ginseng adventitious roots for RNAi gene expression repression; Figure S7: Annotation and GO categorization of *PgRg1-3* gene; Figure S8: Expression heatmaps of *PgRg1-3* with the key enzyme genes for ginsenoside biosynthesis at different ages of ginseng roots; Table S1: Identification of genomic loci significantly associated with Rg1 content using single-locus models and multiple-locus models; Table S2: Analysis of the ±500 kb regions of the two QTNs identified a total 541 candidate genes for Rg1 biosynthesis; Table S3: The number of genes in 26 expression modules by WGCNA; Table S4: *PgRg1* candidate gene containing SNP/InDel mutations that had significant impacts on Rg1 content and the impacts of their SNPs/InDels on Rg1 content.
