# Supplementary material for: Identification of PgRg1-3 Gene for Ginsenoside Rg1 Biosynthesis as Revealed by Combining Genome-Wide Association Study and Gene Co-Expression Network Analysis of Jilin Ginseng Core Collection
Source: Plants (Basel). 2024 Jun 27;13(13):1784. doi: 10.3390/plants13131784 (PMC11244481; doi:10.3390/plants13131784)
Supplement: Supplementary file 1 [file plants-13-01784-s001.zip › Figure S1_BLUP.pptx]

## Slide 1
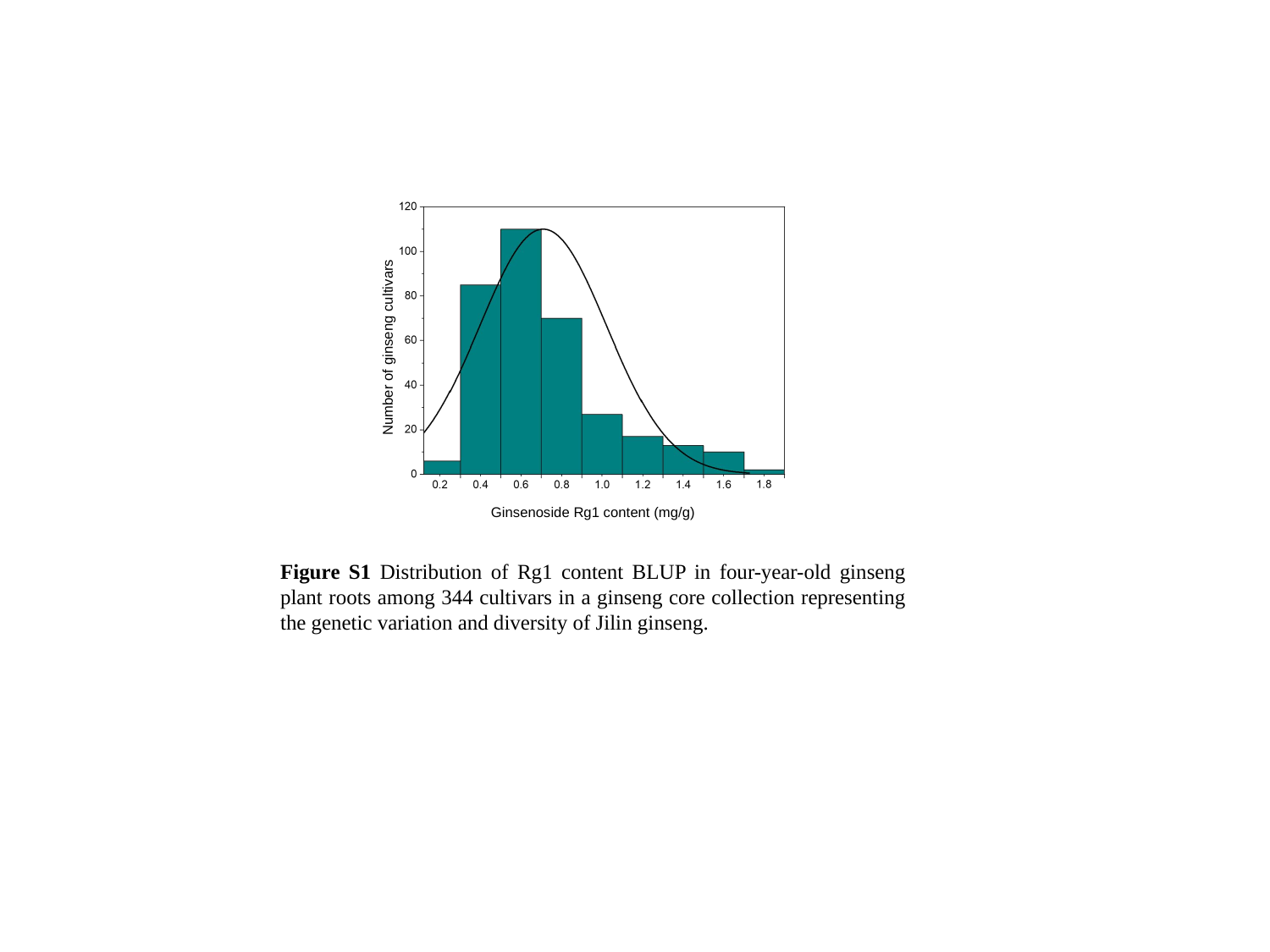

Number of ginseng cultivars
Ginsenoside Rg1 content (mg/g)
Figure S1 Distribution of Rg1 content BLUP in four-year-old ginseng plant roots among 344 cultivars in a ginseng core collection representing the genetic variation and diversity of Jilin ginseng.
