# Supplementary material for: Identification of PgRg1-3 Gene for Ginsenoside Rg1 Biosynthesis as Revealed by Combining Genome-Wide Association Study and Gene Co-Expression Network Analysis of Jilin Ginseng Core Collection
Source: Plants (Basel). 2024 Jun 27;13(13):1784. doi: 10.3390/plants13131784 (PMC11244481; doi:10.3390/plants13131784)
Supplement: Supplementary file 1 [file plants-13-01784-s001.zip › Figure S2_multiple model.pptx]

## Slide 1
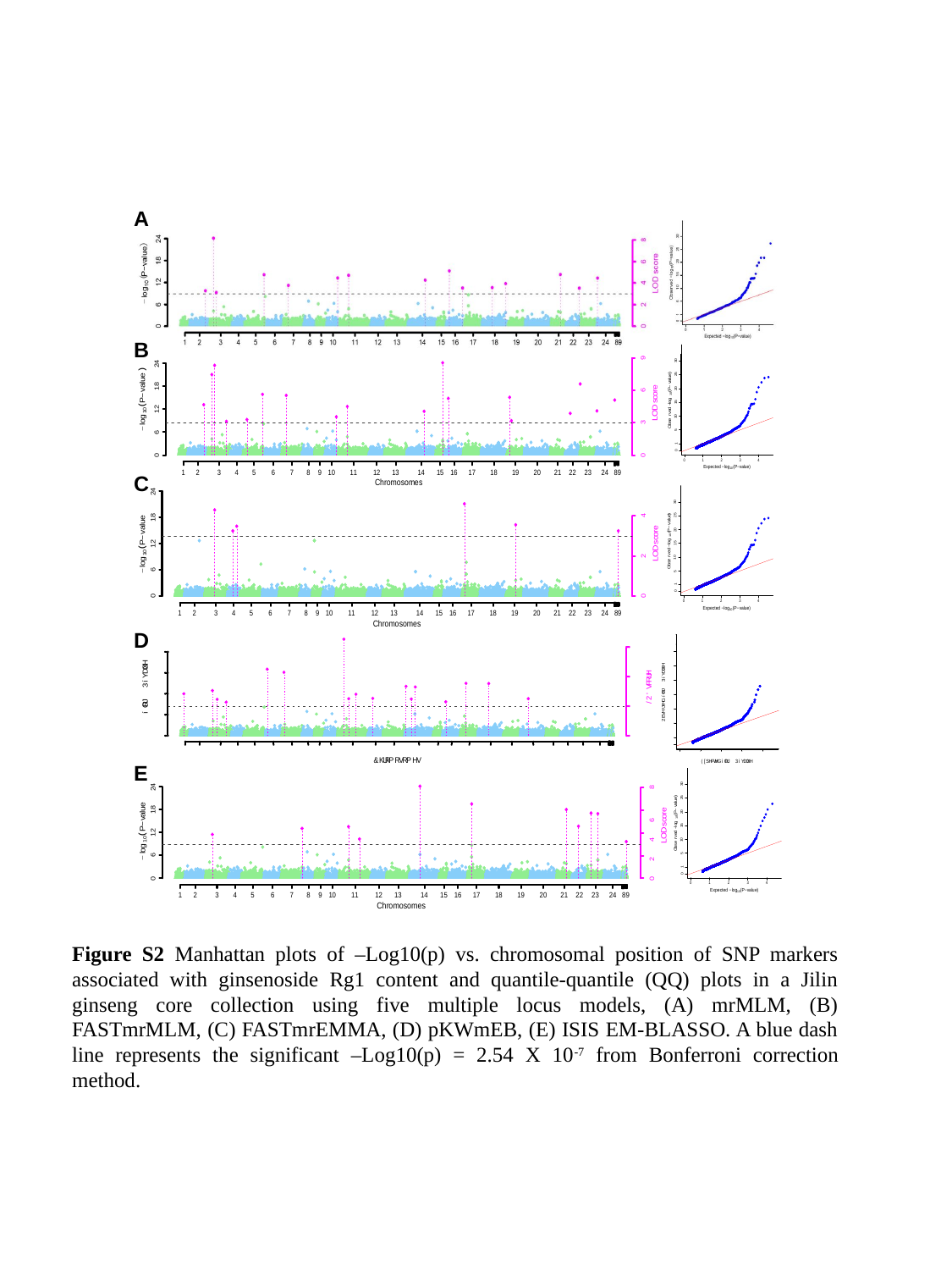

A
B
C
D
E
Figure S2 Manhattan plots of –Log10(p) vs. chromosomal position of SNP markers associated with ginsenoside Rg1 content and quantile-quantile (QQ) plots in a Jilin ginseng core collection using five multiple locus models, (A) mrMLM, (B) FASTmrMLM, (C) FASTmrEMMA, (D) pKWmEB, (E) ISIS EM-BLASSO. A blue dash line represents the significant –Log10(p) = 2.54 X 10-7 from Bonferroni correction method.
