# Supplementary material for: Identification of PgRg1-3 Gene for Ginsenoside Rg1 Biosynthesis as Revealed by Combining Genome-Wide Association Study and Gene Co-Expression Network Analysis of Jilin Ginseng Core Collection
Source: Plants (Basel). 2024 Jun 27;13(13):1784. doi: 10.3390/plants13131784 (PMC11244481; doi:10.3390/plants13131784)
Supplement: Supplementary file 1 [file plants-13-01784-s001.zip › Figure S3_LD.pptx]

## Slide 1
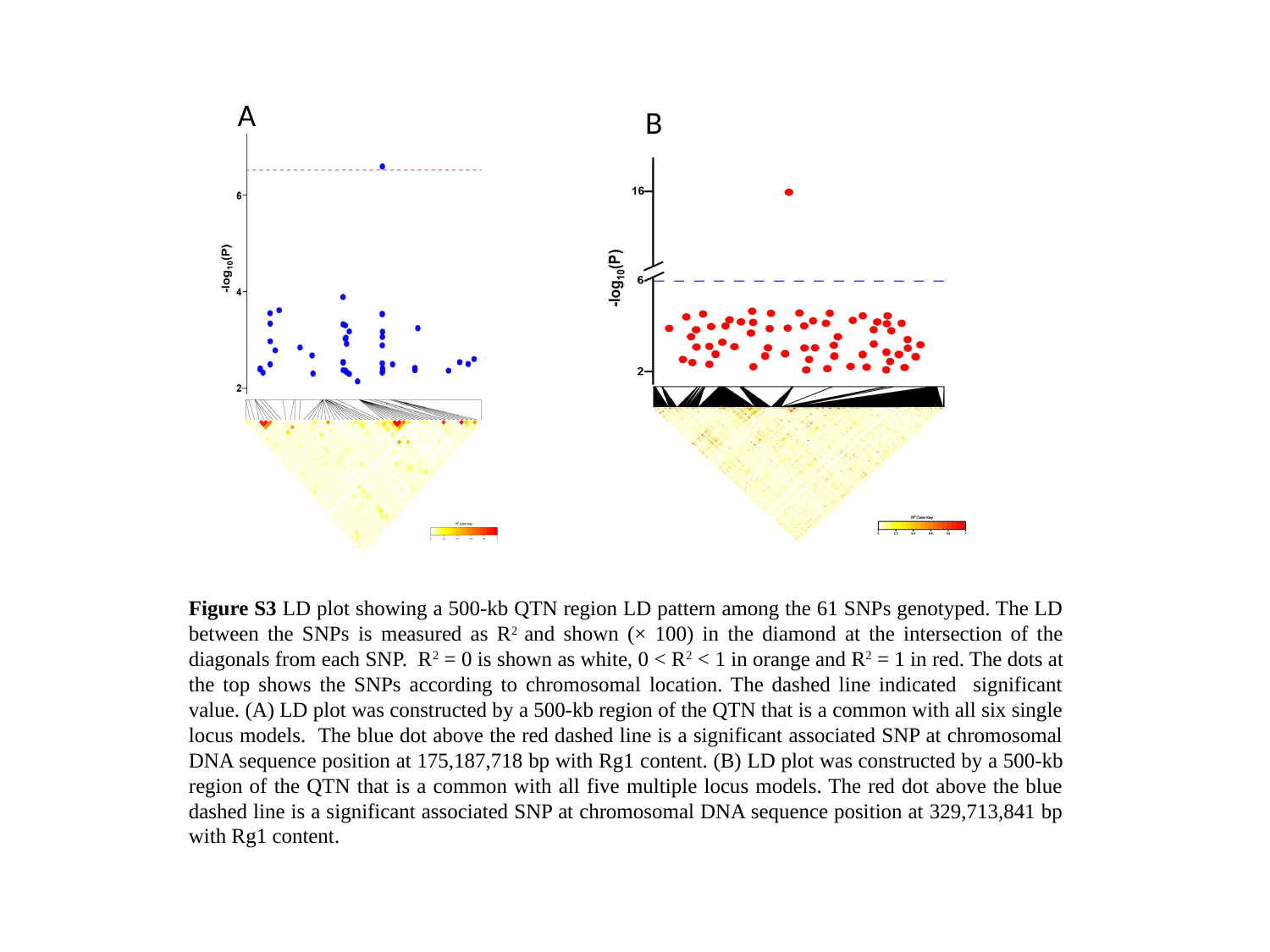

A
B
Figure S3 LD plot showing a 500-kb QTN region LD pattern among the 61 SNPs genotyped. The LD between the SNPs is measured as R2 and shown (× 100) in the diamond at the intersection of the diagonals from each SNP. R2 = 0 is shown as white, 0 < R2 < 1 in orange and R2 = 1 in red. The dots at the top shows the SNPs according to chromosomal location. The dashed line indicated significant value. (A) LD plot was constructed by a 500-kb region of the QTN that is a common with all six single locus models. The blue dot above the red dashed line is a significant associated SNP at chromosomal DNA sequence position at 175,187,718 bp with Rg1 content. (B) LD plot was constructed by a 500-kb region of the QTN that is a common with all five multiple locus models. The red dot above the blue dashed line is a significant associated SNP at chromosomal DNA sequence position at 329,713,841 bp with Rg1 content.
