# Supplementary material for: Identification of PgRg1-3 Gene for Ginsenoside Rg1 Biosynthesis as Revealed by Combining Genome-Wide Association Study and Gene Co-Expression Network Analysis of Jilin Ginseng Core Collection
Source: Plants (Basel). 2024 Jun 27;13(13):1784. doi: 10.3390/plants13131784 (PMC11244481; doi:10.3390/plants13131784)
Supplement: Supplementary file 1 [file plants-13-01784-s001.zip › Figure S4_WGCNA.pptx]

## Slide 1
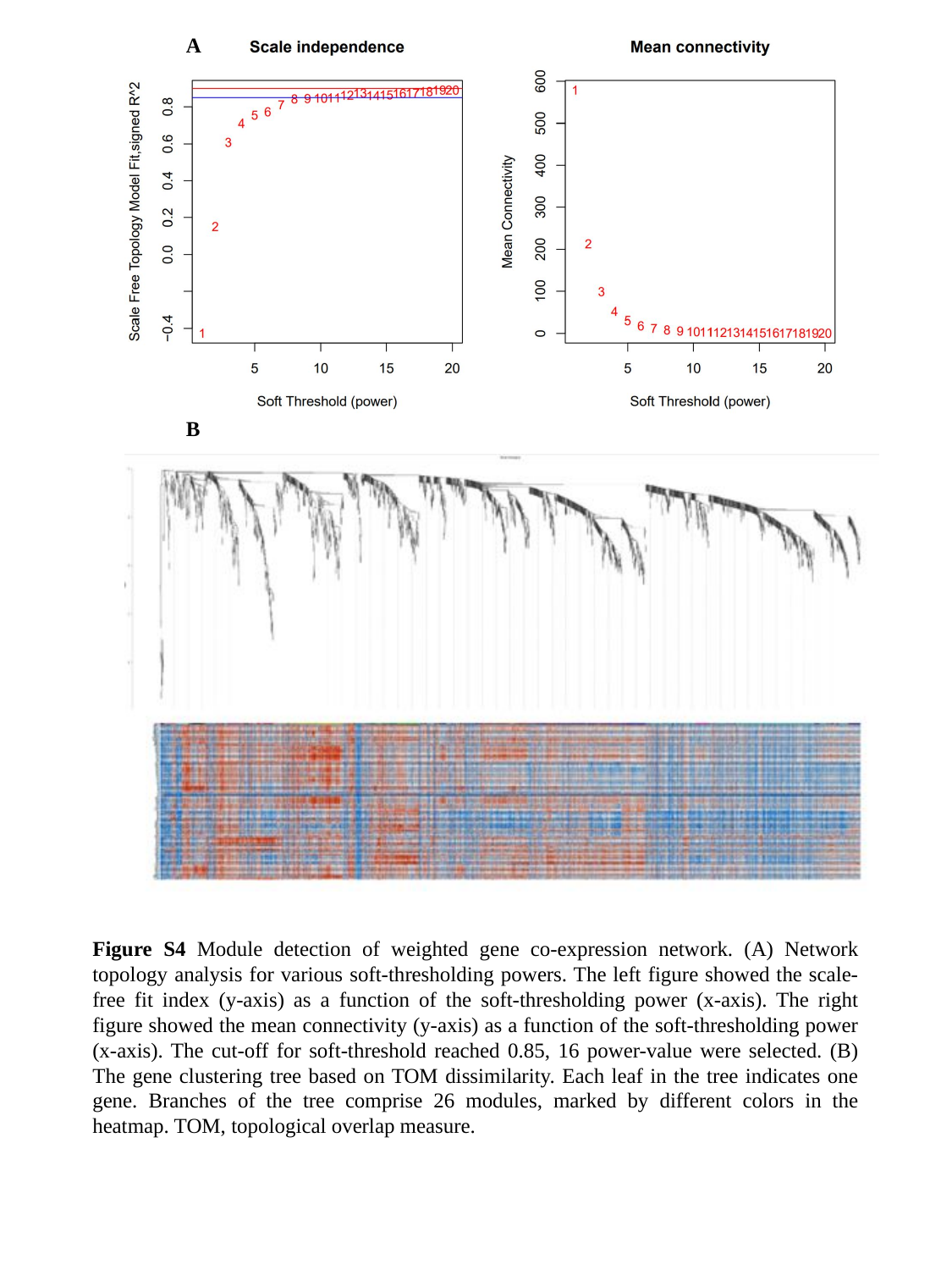

A
B
Figure S4 Module detection of weighted gene co-expression network. (A) Network topology analysis for various soft-thresholding powers. The left figure showed the scale-free fit index (y-axis) as a function of the soft-thresholding power (x-axis). The right figure showed the mean connectivity (y-axis) as a function of the soft-thresholding power (x-axis). The cut-off for soft-threshold reached 0.85, 16 power-value were selected. (B) The gene clustering tree based on TOM dissimilarity. Each leaf in the tree indicates one gene. Branches of the tree comprise 26 modules, marked by different colors in the heatmap. TOM, topological overlap measure.
