# Supplementary material for: Identification of PgRg1-3 Gene for Ginsenoside Rg1 Biosynthesis as Revealed by Combining Genome-Wide Association Study and Gene Co-Expression Network Analysis of Jilin Ginseng Core Collection
Source: Plants (Basel). 2024 Jun 27;13(13):1784. doi: 10.3390/plants13131784 (PMC11244481; doi:10.3390/plants13131784)
Supplement: Supplementary file 1 [file plants-13-01784-s001.zip › Figure S5_key enzyme genes indeced by MeJA.pptx]

## Slide 1
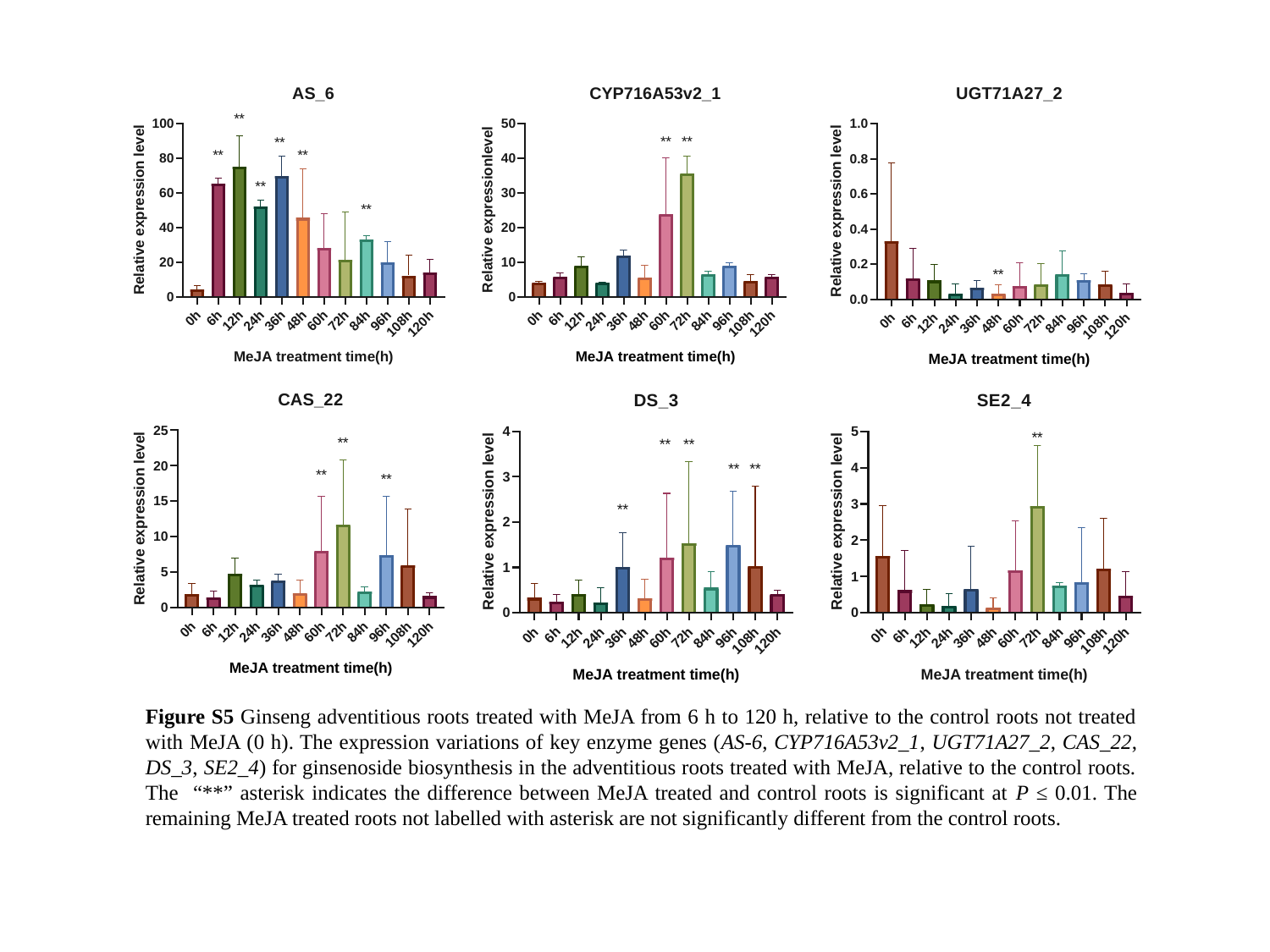

Figure S5 Ginseng adventitious roots treated with MeJA from 6 h to 120 h, relative to the control roots not treated with MeJA (0 h). The expression variations of key enzyme genes (AS-6, CYP716A53v2_1, UGT71A27_2, CAS_22, DS_3, SE2_4) for ginsenoside biosynthesis in the adventitious roots treated with MeJA, relative to the control roots. The “**” asterisk indicates the difference between MeJA treated and control roots is significant at P ≤ 0.01. The remaining MeJA treated roots not labelled with asterisk are not significantly different from the control roots.
