# Supplementary material for: Identification of PgRg1-3 Gene for Ginsenoside Rg1 Biosynthesis as Revealed by Combining Genome-Wide Association Study and Gene Co-Expression Network Analysis of Jilin Ginseng Core Collection
Source: Plants (Basel). 2024 Jun 27;13(13):1784. doi: 10.3390/plants13131784 (PMC11244481; doi:10.3390/plants13131784)
Supplement: Supplementary file 1 [file plants-13-01784-s001.zip › Figure S6_hairy roots.pptx]

## Slide 1
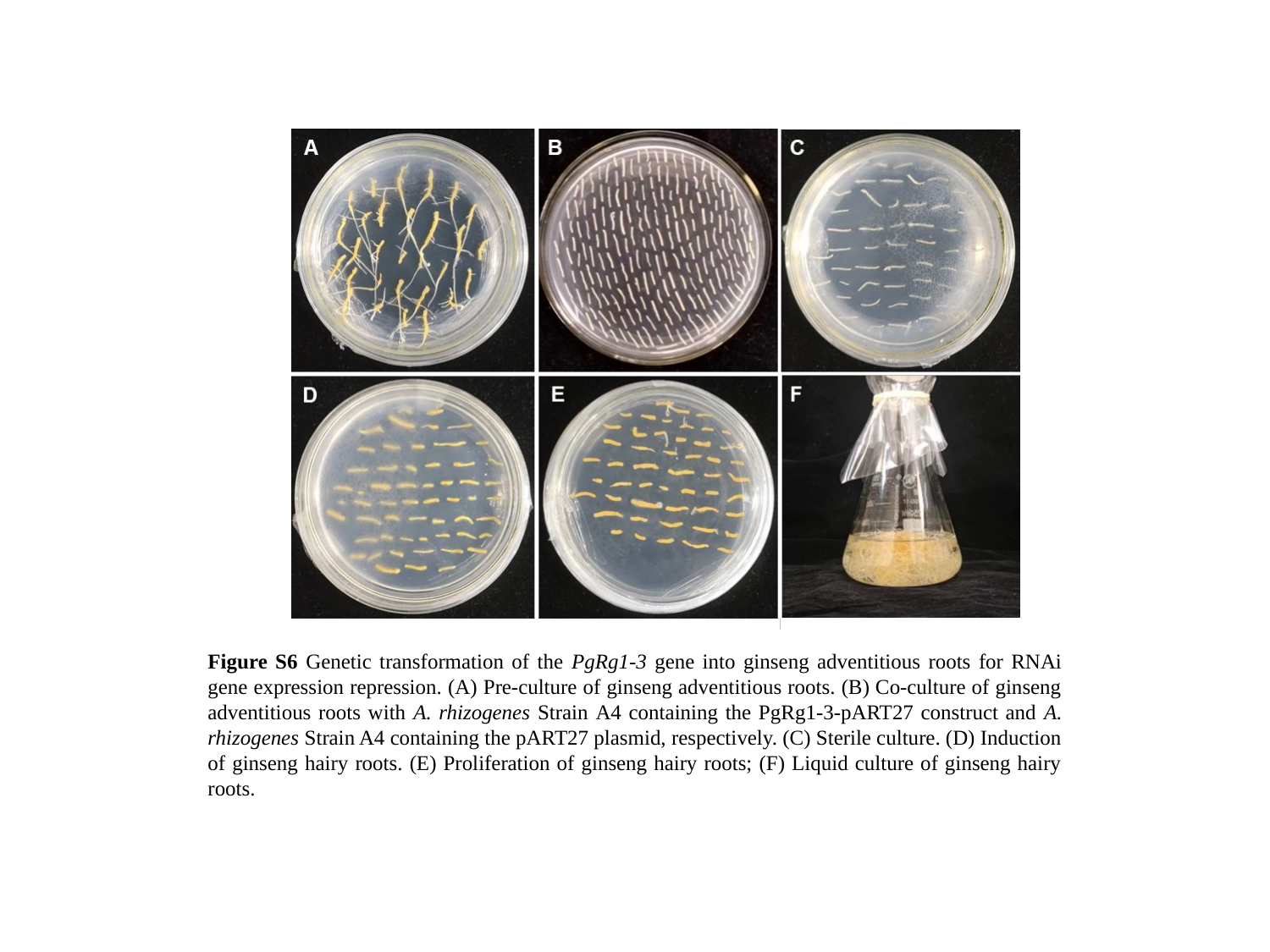

Figure S6 Genetic transformation of the PgRg1-3 gene into ginseng adventitious roots for RNAi gene expression repression. (A) Pre-culture of ginseng adventitious roots. (B) Co-culture of ginseng adventitious roots with A. rhizogenes Strain A4 containing the PgRg1-3-pART27 construct and A. rhizogenes Strain A4 containing the pART27 plasmid, respectively. (C) Sterile culture. (D) Induction of ginseng hairy roots. (E) Proliferation of ginseng hairy roots; (F) Liquid culture of ginseng hairy roots.
