# Supplementary material for: Identification of PgRg1-3 Gene for Ginsenoside Rg1 Biosynthesis as Revealed by Combining Genome-Wide Association Study and Gene Co-Expression Network Analysis of Jilin Ginseng Core Collection
Source: Plants (Basel). 2024 Jun 27;13(13):1784. doi: 10.3390/plants13131784 (PMC11244481; doi:10.3390/plants13131784)
Supplement: Supplementary file 1 [file plants-13-01784-s001.zip › Figure S7_GO and ortholog gene alignment.pptx]

## Slide 1
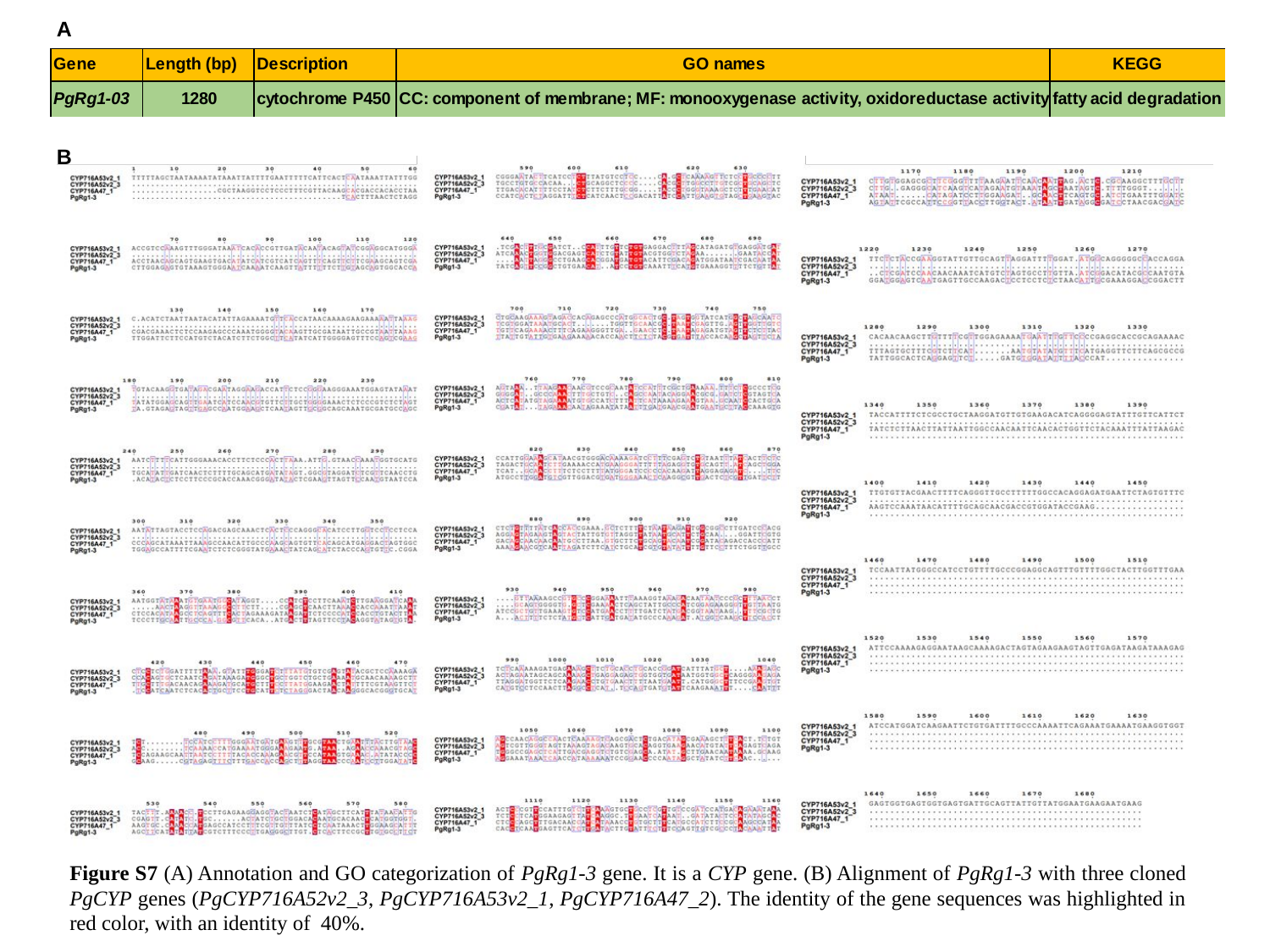

A
B
Figure S7 (A) Annotation and GO categorization of PgRg1-3 gene. It is a CYP gene. (B) Alignment of PgRg1-3 with three cloned PgCYP genes (PgCYP716A52v2_3, PgCYP716A53v2_1, PgCYP716A47_2). The identity of the gene sequences was highlighted in red color, with an identity of 40%.
