# Supplementary material for: Identification of PgRg1-3 Gene for Ginsenoside Rg1 Biosynthesis as Revealed by Combining Genome-Wide Association Study and Gene Co-Expression Network Analysis of Jilin Ginseng Core Collection
Source: Plants (Basel). 2024 Jun 27;13(13):1784. doi: 10.3390/plants13131784 (PMC11244481; doi:10.3390/plants13131784)
Supplement: Supplementary file 1 [file plants-13-01784-s001.zip › Figure S8_Heatmap.pptx]

## Slide 1
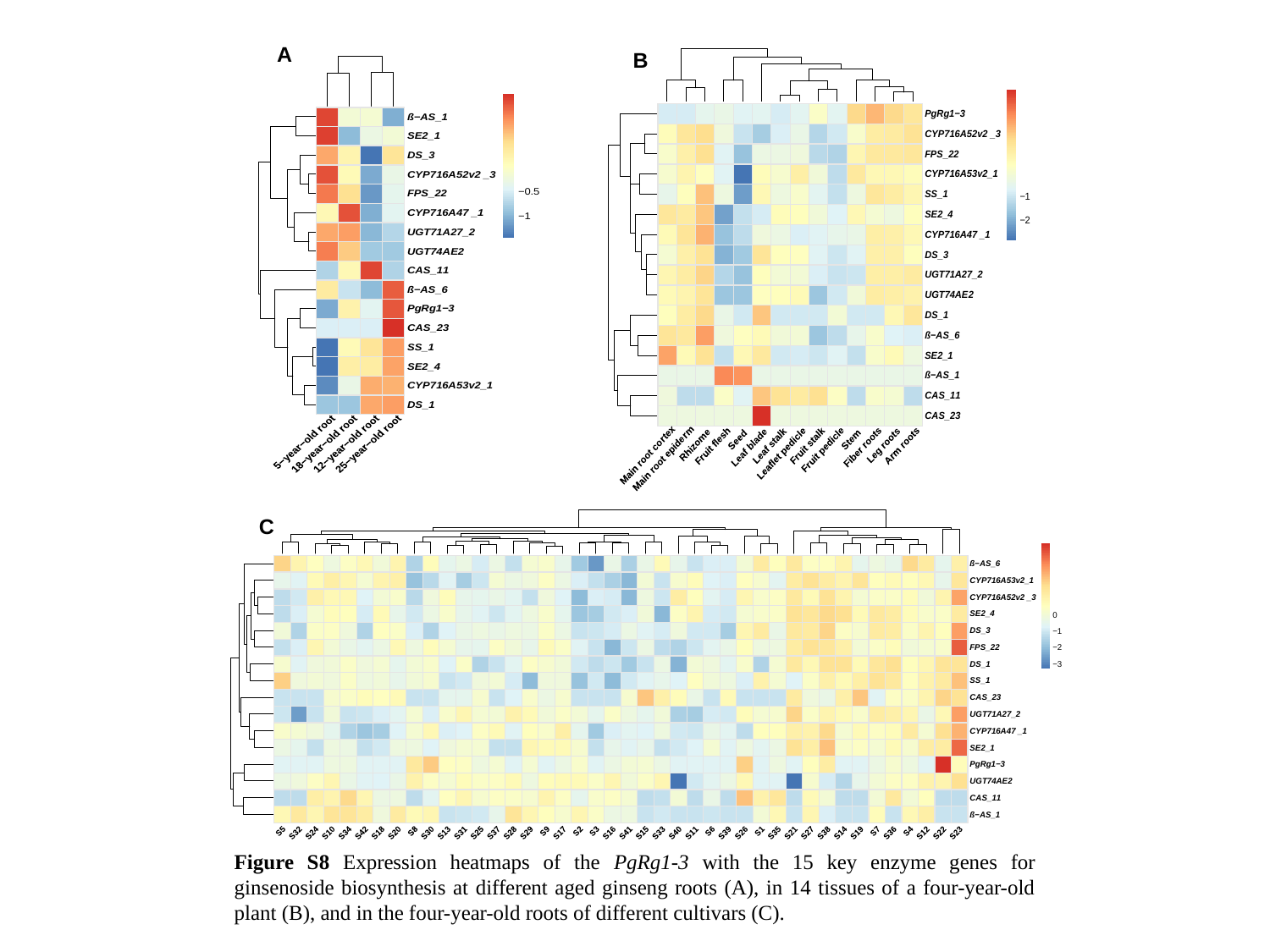

A
B
C
Figure S8 Expression heatmaps of the PgRg1-3 with the 15 key enzyme genes for ginsenoside biosynthesis at different aged ginseng roots (A), in 14 tissues of a four-year-old plant (B), and in the four-year-old roots of different cultivars (C).
